# Identifying the drivers and trends of Iran nursing education: a multi-methods study

**DOI:** 10.1186/s12912-025-03169-8

**Published:** 2025-07-01

**Authors:** Mahboubeh Shali, Alireza Nikbakht Nasrabadi, Azam Ghorbani, Neda Sheikhzakaryaee, Marzieh Sobhani, Atefeh Vaezi, Mansoureh Sepehrinia

**Affiliations:** 1 Critical Care Nursing Department, School of Nursing and Midwifery, Tehran University of Medical Sciences, Tehran, Iran; 2 Medical Surgical Nursing Department, School of Nursing and Midwifery, Tehran University of Medical Sciences, Tohid Square, Tehran, 1419733171 Iran; 3 Nursing and Midwifery School, Kurdistan University of Medical Sciences, Sanandaj, Iran; 4 Nursing Department, Dehaghan Islamic Azad University, Dehaghan, Iran; 5 Department of Pediatric Nursing, School of Nursing and Midwifery, Zanjan University of Medical Sciences, Zanjan, Iran

**Keywords:** Nursing education, Drivers, Meta-synthesis, Shannon’s entropy, Delphi

## Abstract

**Introduction:**

Nursing education faces many challenges that can be solved with simple and low-cost measures if properly identified, otherwise these challenges will turn into crisis and their solution will be difficult and costly. Proponents or derivers carry out the task of advancing and solving challenges at the same time.

**Objective:**

This study was conducted with the aim of identifying the drivers and trends of nursing education.

**Method:**

The research is developmental-applicative in terms of its purpose and descriptive-exploratory in terms of its nature, and a multi method was used to collect data. In the first stage, 723 articles published between 1990 and 2023 were examined using meta-synthesis. In the next stage, 23 semi-structured interviews were conducted with 17 nursing professors. Shannon’s entropy was used to analyze the content of obtained codes and then, concepts were ranked according to their weights. In the last step, the Delphi method was used to create an agreement.

**Findings:**

In the first stage, after reviewing 9 related articles, 24 codes obtained. After analyzing the interviews, 365 codes identified. The experts agreed on 6 drivers and 31 trends. Empowering policies (such as drivers of using internet and designing systems with greater flexibility), structural and managerial policies (such as drivers of improving management methods/strategies and quality assurance), trans-organizational policies (such as driver of being on the path of globalization), and value policies (such as derivers of developing moral and culture-centeredness) are among the findings of present study.

**Conclusion:**

In order to control the challenges of nursing education and prevent them from turning into crisis, operational and basic solutions can be used to improve curriculum in accordance with global standards and advances in knowledge and technology. It is also suggested to improve the student/tutor ratio, establish an accreditation body at the national level, and allocate performance-based budget for the qualitative development of education system.

**Supplementary Information:**

The online version contains supplementary material available at 10.1186/s12912-025-03169-8.

## Introduction

One of the most important missions of higher education (university) system in nursing faculties is to empower students to accept the important roles of nursing profession [1]. The purpose of nursing education in nursing schools is to create critical and creative thinking, promote self-directed learning, improve psychomotor skills and time management ability, increase self-confidence, establish proper communication and prevent students’ passivity [2]. The university system should educate experienced nurses with acceptable scientific and practical ability in providing quality care to members of society. At the same time, the need to improve quality, the rapid development of technology and the increase in complexity of the health and treatment system make it necessary to constantly review and revise the academic system [3].

Studies show that the output of nursing schools is not aligned with their goals in educating students [4–6]. As a center for training efficient human resources in the field of health, nursing schools should imagine a better future for nursing and improve their performance by using experiences and reference documents. Nursing managers can seek to find their place and contribution in the future of this changing world by using their vision of future with flexibility and active adaptability [7].

Nursing education, due to its vastness and importance, has very important and complex elements, and neglecting to see future changes, can have irreparable effects on goals that societies intend to achieve. Therefore, finding challenges and recognizing them is of particular importance. Nursing education faces many challenges that can be solved with simple and low-cost measures if properly identified, otherwise they will turn into crisis and their solution will be difficult and costly. Proponents or derivers carry out the task of advancing and solving challenges at the same time. For its development and sustainability, nursing education needs various drivers to help it maintain its existence in this turbulent era.

Drivers are a set of future-shaping forces that affect different futures globally or locally. Drivers indirectly affect the social, technological, economic, environmental and political factors. Drivers consist of several processes that cause change in a studied field. Trends are usually somewhat gradual forces, or in other words, factors and patterns that cause widespread changes in society. The speed of changes may seem relatively slow or fast, but the most important aspect of trends is their pervasiveness [8].

Nursing education in Iran, while having many challenges and crises, has some diverse and somewhat unknown capacities. These drivers and trends, if identified and applied, can overcome some of the existing challenges of nursing education in Iran. Accordingly, this study was conducted to systematically investigate the key drivers and emerging trends influencing nursing education, aiming to provide a comprehensive understanding that can guide the development of effective educational strategies and policies within the nursing profession in Iran.

## Method

The meta-synthesis approach, interview and Delphi methods were used in this study.

### Qualitative meta-synthesis stage

In the first phase of the study, a qualitative meta-synthesis was carried out, which was focused on qualitative studies to create comprehensive and interpretive findings. For this purpose, the seven-step of Sandelowski and Barroso’s method was used [9], which included setting the research question, reviewing the literature in a systematic way, searching and selecting suitable articles, extracting information from the articles, analyzing and combining the findings, checking the quality of findings and presenting them. All the steps of searching the articles were done by the first author.

#### Set research questions

The research question at this stage was designed as follows: “What are the main drivers and trends of nursing education in Iran?”

#### Systematic literature review

The second stage is the systematic review of the literature, at this stage the researcher focuses his systematic search on published papers in various journals and selects keywords to search papers. all relevant qualitative articles published between 1990 and 2023 available at databases such as PubMed, CINAHL, Science Direct, SID, and Magiran were searched. Keywords such as nursing education, higher education, nursing education policy, drivers and trend in Farsi and English in the title and abstract of the articles were used in the search. Boolean operators were nursing education OR higher education OR nursing education policy AND drivers OR trend, transform*nurs*educa*,future AND nurs*educa* OR nurs*education policy. The inclusion criteria for articles included in the study was: Literature published between 1990 and 2023, grey literature, Literature needed to provide an answer to the research questions, Studies using qualitative designs, Studies using structured or unstructured in-depth interviews, Literature written in English or persian language. The exclusion criteria for articles included in the study was: Secondary data analysis and Lack of access to the full text of the article.

#### Searching and selecting suitable articles

**Fig. 1:**
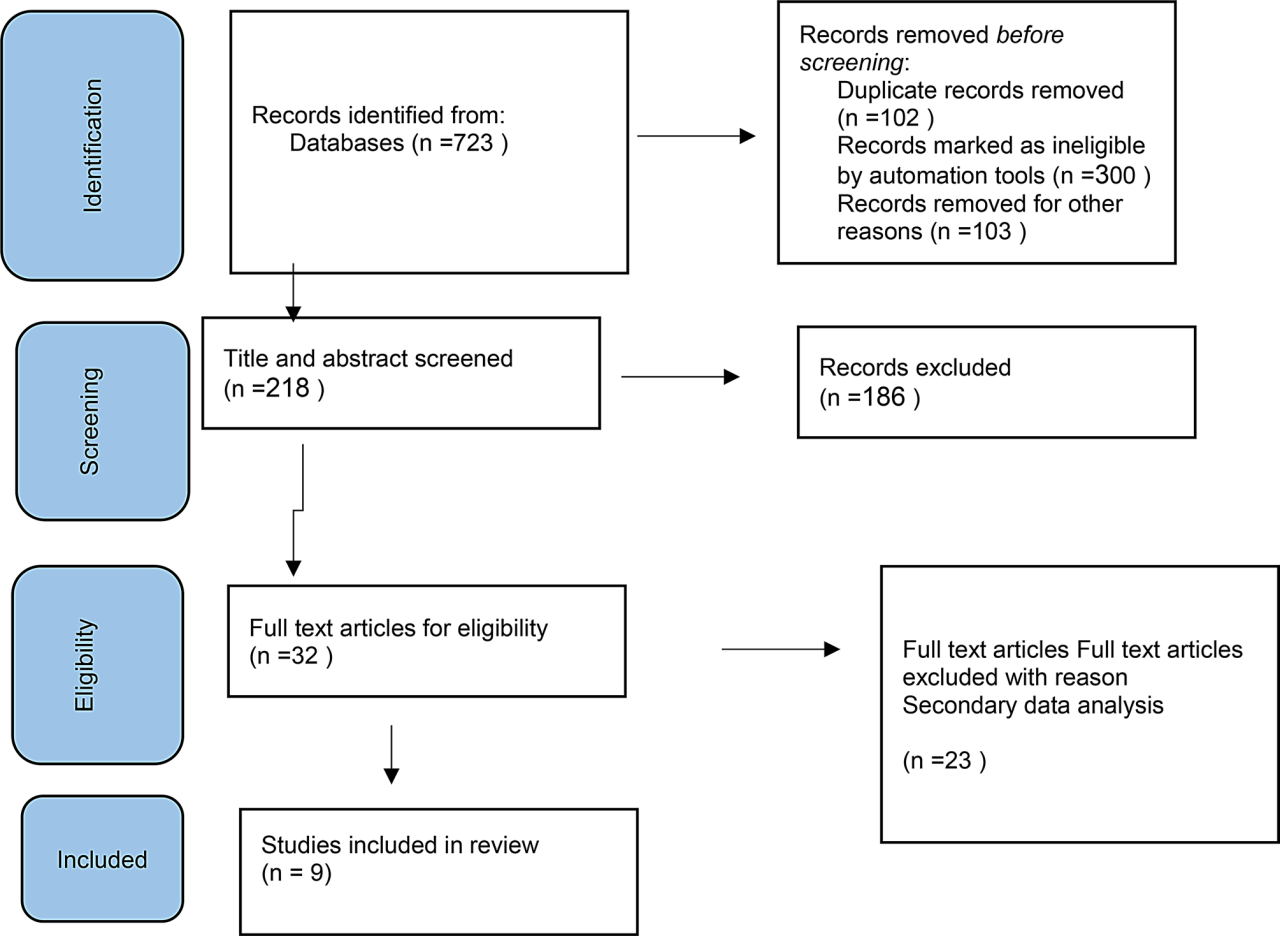
PRISMA flow diagram. Flow of reports from identification to inclusion

After extensive research, the papers found during several stages are reviewed and excluded until appropriate papers are identified and separated from other studies collected. The articles were evaluated in terms of title, abstract, content and quality of research method based on a PRISMA checklist (Fig. 1).

#### Extracting information from the articles

At this stage, the researcher reviews various and final papers continuously (in order to achieve the content results). Then, researcher classify, summarize, and encode obtained information from the references on the basis of criteria such as: the study purpose, and the method and classification of being analyzed.

#### Analyzing and combining the findings

The researcher in this section after identifying subjects, classifies them and puts similar and related categories in a subject that best describe it. At the end of this stage, the researcher identified 127 codes, which were merged together and given common names according to the purpose of study as well as their similarities and differences. At the end of this stage, the number of codes was reduced to 24 codes.

#### Checking the quality of findings

In order to ensure the quality of results, they were given to an expert to review them by Kappa index. Using SPSS software version 16 at a significance level of 0.05, the coefficient of 0.83 was obtained in this study, which shows the appropriate reliability of the codes.

#### Findings presentation

At the final stage of meta synthesis, the results are presented (Table 1).

**Table 1:** The importance coefficient and ranking of drivers and trends of nursing education in Iran

No	Area	Driver	Trends (subordinates of each driver)	Frequency	$$\begin{gathered}\sum\nolimits_i^m {} \hfill \\\left[ {{P_{ij}}\,In{p_{ij}}} \right] \hfill \\ \end{gathered} $$	Lack of confidence	Importance coefficient	Ranking in concept	Ranking in whole
1	Trans-organizational policies	Being in the path of globalization	Providing facilities for study opportunities	11	99/1	93/0	054/0	1	9
2			Participation in the production of international science (Bohman & Borglin, 2014)	4	98/0	35/0	056/0	3	13
3			Guidance and management of the competitiveness between universities(Patterson & Krouse, 2015)	10	93/1	98/0	045/0	2	10
4			Admission and education of foreign students (Bohman & Borglin, 2014)	4	98/0	35/0	056/0	3	13
5			Building trust in relationships between universities (Patterson & Krouse, 2015)	12	11/2	78/0	019/0	2	8
6	Empowering polices	Using interment	The use of telehealth in education	23	51/3	23/1	049/0	1	1
7			Wide use of electronic learning and distance education (Moule, Ward, & Lockyer, 2011)	23	51/3	23/1	049/0	1	1
8			Stabilizing the position of nursing informatics (Moule et al., 2011)	21	89/2	85/0	033/0	2	2
9		Designing system with greater flexibility	The independence of local resources in providing curriculum (Griffiths, Speed, Horne, & Keeley, 2012; Moule et al., 2011)	13	23/2	32/1	087/0	2	7
10			Using the experiences of other universities (Bohman & Borglin, 2014)	9	87/1	65/0	032/0	5	11
11			Diversifying university programs as much as possible (Moule et al., 2011; Muraraneza & Mtshali, 2020)	4	98/0	35/0	056/0	5	13
12			Planning based on the current problems of society (Goddard, Mackey, & Davidson, 2010; Griffiths et al., 2012; Theobald & Ramsbotham, 2019)	15	31/2	93/0	024/0	3	6
13			Making education a problem-centered issue (Goddard et al., 2010; Moule et al., 2011; Muraraneza & Mtshali, 2020; Stenberg, Bengtsson, Mangrio, & Carlson, 2020; Theobald & Ramsbotham, 2019)	19	98/2	88/0	087/0	1	3
14			Increasing change spirit and reducing resistances (Griffiths et al., 2012)	12	11/2	78/0	019/0	4	8
15	Structural and managerial policies	Improving management methods and strategies	Increasing interdisciplinary concepts (Theobald & Ramsbotham, 2019)	13	23/2	32/1	087/0	4	7
16			Result-centered management (Patterson & Krouse, 2015)	4	98/0	35/0	056/0	4	13
17			Openness and honesty in policies and practices (Patterson & Krouse, 2015)	10	93/1	98/0	045/0	6	10
18			Development of organizational learning and systemic thinking (Patterson & Krouse, 2015; Theobald & Ramsbotham, 2019)	4	98/0	35/0	056/0	6	13
19			Using adequate and appropriate human resources (Moule et al., 2011; Theobald & Ramsbotham, 2019)	15	31/2	93/0	024/0	1	6
20			Increasing education while serving teachers (Griffiths et al., 2012; Moule et al., 2011)	8	53/1	12/1	056/0	4	12
21			Creating investment opportunities in education (Moule et al., 2011; Muraraneza & Mtshali, 2020)	21	12/3	98/0	078/0	1	2
22			Clarifying the roles and responsibilities of tutors (Theobald & Ramsbotham, 2019)	15	31/2	93/0	024/0	3	5
23			Appropriate distribution of funds in education	16	42/2	97/0	078/0	2	5
24			Creating educational opportunities for entrepreneurship in nursing	13	23/2	32/1	087/0	4	7
25			Mission-centeredness (Muraraneza & Mtshali, 2020; Patterson & Krouse, 2015)	4	98/0	35/0	056/0	6	13
26		Quality assurance	Establishment of an accreditation body at the national level	12	11/2	78/0	019/0	2	8
27			Use of quality control measures	18	89/2	85/0	033/0	1	4
28			Changing the method of student assessment from quantitative to qualitative assessment (Goddard et al., 2010; Muraraneza & Mtshali, 2020; Theobald & Ramsbotham, 2019)	8	53/1	12/1	056/0	3	12
29	Value policies	Improving moral and culture-centeredness	The prevalence and development of intercultural learning(Bohman & Borglin, 2014; Moule et al., 2011; Patterson & Krouse, 2015)	9	87/1	65/0	032/0	3	11
30			Promoting cultural competence in education (Bohman & Borglin, 2014; Moule et al., 2011; Stenberg et al., 2020)	15	31/2	93/0	024/0	2	6
31			Educating students who are sensitive to professional ethics	19	98/2	88/0	087/0	1	3

References

Bohman, D. M., & Borglin, G. (2014). Student exchange for nursing students: Does it raise cultural awareness’? A descriptive, qualitative study. *Nurse Education in Practice*,* 14*(3), 259–264

Goddard, L., Mackey, S., & Davidson, P. M. (2010). Functional clinical placements: a driver for change. *Nurse education today*,* 30*(5), 398–404

Griffiths, J., Speed, S., Horne, M., & Keeley, P. (2012). ‘A caring professional attitude’: What service users and carers seek in graduate nurses and the challenge for educators. *Nurse Education Today*,* 32*(2), 121–127

Moule, P., Ward, R., & Lockyer, L. (2011). Issues with e-learning in nursing and health education in the UK: are new technologies being embraced in the teaching and learning environments? *Journal of Research in Nursing*,* 16*(1), 77–90

Muraraneza, C., & Mtshali, N. G. (2020). Drivers of transformation to competency-based nursing education in Rwanda. *International Journal of Africa Nursing Sciences*,* 13*, 100,224

Patterson, B. J., & Krouse, A. M. (2015). Competencies for leaders in nursing education. *Nursing Education Perspectives*,* 36*(2), 76–82

Stenberg, M., Bengtsson, M., Mangrio, E., & Carlson, E. (2020). Preceptors’ experiences of using structured learning activities as part of the peer learning model: A qualitative study. *Nurse education in practice*,* 42*, 102668

Theobald, K. A., & Ramsbotham, J. (2019). Inquiry-based learning and clinical reasoning scaffolds: An action research project to support undergraduate students’ learning to ‘think like a nurse’. *Nurse education in practice*,* 38*, 59–65

### Interview stage

Qualitative interviews were conducted during 4 months (August- November 2023) in the following steps.

#### Sample and setting

Sampling was done purposively and with maximum variation in terms of participants’ gender, educational level in all nursing education fields, work experience, and work environment. The key informants included nursing professors and tutors from all over the country. Inclusion criteria were including teaching and management experience in the field of nursing education, agreement for participation in the study, and ability to share personal experiences.

#### Data collection

In this step, semi-structured interviews were conducted. All interviews were conducted by the corresponding author. The main question asked from the participants was: “What are the driving forces and trends of nursing undergraduate education in your opinion?” In order to advance the interviews, exploratory questions were also used to clear any ambiguities (Appendix 1). Interviews were conducted via phone and in person. Interviews were held at participants’ preferred time and place and lasted 40 min. Data collection lasted for 4 months until reaching data saturation. Study data were saturated after the 20 interview; yet, 3 more interviews were done to ensure data saturation. Interviews were digitally recorded. In conducting all the interviews, the principle of bracketing (looking at a situation and refraining from judgement and biased opinions to wholly understand an experience) was considered and interviews were conducted by another researcher.

#### Data analysis

Shannon’s entropy method was used to analyze the content of the codes [10]. First, the message was counted as frequency for each respondent, and then the importance of each code was calculated using the information load of that code. Based on this, the consistency of past studies and the findings of this study can be shown statistically. Equations 1 and 2 were used to calculate the information load of “lack of confidence” and “importance coefficient”. To calculate the weight of each concept, the total weight of the codes related to that concept was calculated and based on the obtained weights, the ranking was done (Eqs. 1, 2) (Appendix 2). Code analysis at this stage was done by the first author.

#### Trustworthiness

Trustworthiness was applied using the criteria proposed by Guba and Lincoln, namely credibility, dependability, confirmability, and transferability [11].

### Delphi process

In the next step, the Delphi method was used to confirm and evaluate the identified drivers. The reason for using Delphi method was the difficulty or impossibility of gathering the specialists in one place, and also the importance of their views and opinions about the drivers and trends of nursing education in Iran.

#### Research community

The research community included nursing specialists (members of the nursing board, educational directors and faculty members of all nursing & midwifery schools in Iran). The people who participated in the interview were not used in the Delphi process, so that the experiences of more people could be used in this study. Using the purposeful sampling method, a total sample size was determined to be 38 people.

#### Delphi technique

A report of the drivers and trends identified in the previous stage along with a summary of study method and objectives were sent to the participants. The scoring range of 0 to 5 was used in the evaluation process, which continued in three consecutive rounds in the form of in-person and via e-mail. Scores of 4 and 5 were considered as good scores.

### Ethics approval and consent to participate

This study was conducted with the permission of Research Ethics Committee of National Center for Strategic Research in Medical Education (Iran). All the participants signed the informed consent form after being informed about the objectives and method of the study. Maintaining the confidentiality of information and having the right to withdraw from the study at any time were among ethical principles observed in this study.

### Findings

The three stages of research are presented in order below.

### Qualitative meta-synthesis stage

Among the 723 qualitative articles that were reviewed, nine articles were used for the final analysis. At the end of this stage, the researcher identified 127 primary codes. Similar codes were merged and duplicate codes were removed. Finally, 24 codes were obtained. In order to evaluate the quality, the results were given to one of the experts to be evaluated by the Kappa index. Using SPSS version 16 software, at a significance level of 0.05, this coefficient was obtained as 0.83, which shows the appropriate reliability of the codes.

### Interview stage

In the interview phase, 17 nursing tutors (2 professors, 6 associate professors, 6 assistant professors, and 3 instructors) with the mean clinical experience of 16 ± 1.2 years and mean managerial experience of 6 ± 3.1 years were interviewed. A total of 23 interviews were conducted that on average took about 25–30 min each. To obtain more information, 6 participants were interviewed twice. Also, 15 of the interviews were conducted over the phone and the rest were conducted face-to-face. In this stage, 356 codes were obtained.

- **Shannon’s entropy results**.

Many of the codes obtained from the interview phase overlapped with the codes obtained from the meta-synthesis stage. The frequency of repetition of each code in both stages (meta-synthesis and interview) is shown in Table 1. The findings of content analysis using Shannon’s entropy in relation to the trans-organizational policies, empowering policies, structural and managerial policies and value policies are shown in Table 1.

### Delphi process

**Fig. 2:**
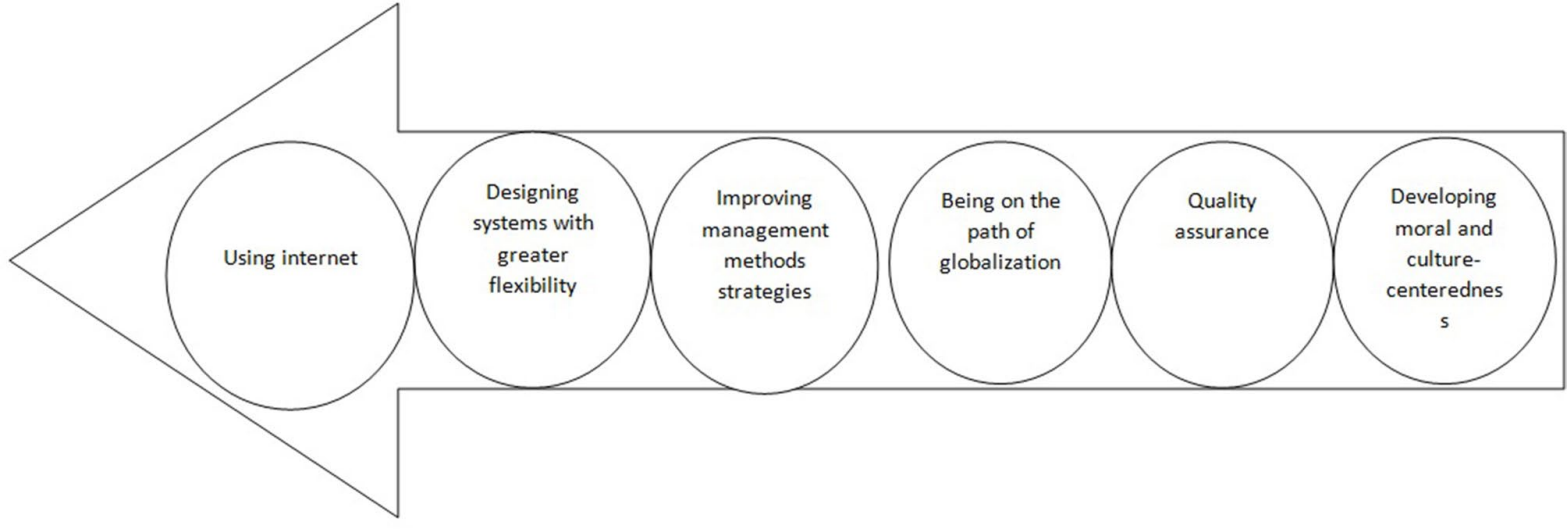
Drivers of nursing education in Iran according to their priority from the experts’ point of view

In this stage, experts agreed on six main drivers and 31 trends in Iran nursing education, as well as their importance. Kendall’s coefficient of agreement was used to calculate the degree of agreement among samples in regards to the drivers and trends. Kendall’s coefficient of agreement for the responses of samples in the third round was 0.82, which indicates a strong agreement among the samples. Empowering policies with the drivers of using internet and designing systems with greater flexibility, structural policies with the drivers of improving management methods/strategies and quality assurance, trans-organizational policies with the driver of being on the path of globalization, and value policies with the drivers of developing moral and culture-centeredness were introduced as drivers of nursing education in Iran, as shown in Fig. 2.

#### Empowering Polices (Using internet)

Empowering policies such as using internet, was one of the important drivers that was achieved with Shannon’s entropy. Wide use of electronic learning and distance education and Stabilizing the position of nursing informatics were trends mentioned in the literature review for Internet use. The “use of telehealth in education” was another trend that emerged from the analysis of the interviews. One nursing professor shared her experience on the importance of using the Internet and telehealth in nursing education:

Students, learners, patients, and ultimately society are looking for convenience and time savings. Technology is constantly advancing. We need to be on the path to progress by incorporating it into nursing education. (P 2).

#### Empowering Polices (Designing system with greater flexibility)

Designing system with greater flexibility was one of the key drivers in the Empowering policies that was introduced. To achieve this driver, following the trends of local resource independence in curriculum provision, using the experiences of other universities, increasing interdisciplinary concepts, and creating diversity in university courses can be successful policies. Making education a problem-centered issue was an important trend that was heavily emphasized in both the interview analysis and the literature review. A nursing professor with 20 years of experience teaching theoretical and clinical nursing emphasized:

Through education based on current societal problems, we can strengthen students in problem solving. This should be considered as a principle in education when designing educational programs. (P 7)

#### Structural and managerial policies (Improving management methods strategies)

According to the research findings, Improving management methods and strategies is an important driver in structural and management policies. Results-centered management, honesty in policy-making, systems thinking, utilizing adequate and appropriate human resources, increasing in-service training, investing in training, and clearly specifying professors’ job descriptions were among the trends mentioned in the literature review. “Appropriate distribution of funds in education” and “creating educational opportunities for entrepreneurship in nursing” were the trends that emerged from the analysis of the interviews. In this regard, a member of the nursing board with 15 years of experience shared his experience as follows:

However, training and maintaining the educational system is expensive. Without sufficient budget, the set goal cannot be achieved. The financial infrastructure must be reformed. The distribution of budget must be properly managed. Financial balance is an important issue that cannot be ignored. (P 3)

#### Structural and managerial policies (Quality assurance)

Based on research findings Quality assurance is another driver in the Structural and managerial policies area. Changing the method of student assessment from quantitative to qualitative assessment was a trend that was mentioned in several studies to achieve quality assurance in education. Based on the analysis of the experiences of the professors participating in the research “Establishment of an accreditation body at the national level” and “Use of quality control measures” are two important trends emerged from the analysis of the interviews.

In this regard, a nursing professor with 4 years of experience as a dean of the Faculty of Nursing added:

We must have a suitable and comprehensive model for evaluation and validation. This model must be designed in such a way that it can be responsive in any city and with any type of facilities. The model must be implementable and simple so that everyone can use it. (P 13)

#### Trans-organizational policies (Being on the path of globalization)

Being on the path of globalization is a driver is within the area of Trans-organizational policies. Participation in the production of international science, Guidance and management of the competitiveness between universities, Admission and education of foreign students and Building trust in relationships between universities were trends that were identified in the metasynthesis. “Providing facilities for study opportunities” was a trend that derived from interviews with nursing professors. In this regard, a nursing professor with 18 years of teaching experience stated:

By training professors at leading universities, nursing education can be guided in the right direction to a large extent. Having the opportunity to study for professors at leading universities in the world can put nursing education on the path to globalization. (P9)

#### Value policies (Improving moral and culture-centeredness)

In the Value policies area, the driver of Improving moral and culture-centeredness was introduced. In this driver, the trends of the prevalence and development of intercultural learning and promoting cultural competence in education were obtained from the meta synthesis. Based on the analysis of the interviews, the trend of Educating students who are sensitive to professional ethics was introduced. In this regard, a nursing professor with 12 years of teaching experience shared his experience as follows:

Ethics play an important role in nursing care. Nursing students should be educated from the beginning to be sensitive to ethical issues. In this case, we can expect to provide ethical care to the patient. Ethical nursing education is the foundation of ethical care. (P 1)

## Discussion

The present study was conducted with the aim of identifying the drivers and trends of nursing education in Iran. Empowering policies such as using internet and designing systems with greater flexibility obtained the highest score in terms of priority. As the daily use of technology in education becomes more common, students must be prepared to use these technologies effectively. The use of telehealth, distance education and nursing informatics are among factors that have been mentioned in regard to the trends of nursing education. The use of telehealth allows people to have a virtual presence instead of physical presence. It also prevents long distance travel, enables the transfer of large amount of information in a short time, and reduces costs [12, 13]. The basis of any intelligent decision that leads to the realization of goals is access to accurate information [14]. The use of informatics in nursing makes it possible to have access to accurate information. Newbold and Westra considered the features of informatics in nursing and paid attention to them in the form of combined educational program at expert level [15]. In the United States, Warren and colleagues also conducted an online research on these programs to identify the required competencies and obtain sufficient information in nursing informatics [16].

The trends of designing programs based on the society’s needs and making education a problem-centered process have been mentioned in relation to the design of systems with greater flexibility. The problem solving skill is an acquired knowledge that is achieved at high perceptual, cognitive and emotional levels. Therefore, education will have a significant contribution in improving the problem solving skills of learners, especially nursing students [17]. In a comprehensive review, Haji Babaei and Ashrafizadeh reported the effectiveness of method that are based on the problem solving skills and empowering students in nursing education [18]. The need to follow trends requires flexibility in the system. A qualitative study in this field showed that the degree of concentration in management, the role and characteristics of manager, and the changing of employees’ attitude are among important management challenges in nursing schools [19].

The trends of budget al.location and creation of investment opportunities in nursing education have been mentioned in relation to the driver of promoting management methods and strategies. Due to the lack of financial resources and budget as well as the ever-increasing costs, educational organizations are forced to implement new strategies for optimal use of resources and cost management in order to maintain their survival and continuity. This requires awareness and transparency in spending available resources and analyzing it. Due to the fact that in some cases, it is difficult to separate educational and medical expenses, it is important to resort to a mechanism based on which expenses can be calculated with an estimate close to reality, because not paying attention to how financial resources are spent and lack of transparency in this field can threaten the sustainability of nursing education [20]. Considering that the current attention of most universities is on entrepreneurship education as a solution to economic problems and a factor that accelerates economic development, nursing schools are also suggested to initiate entrepreneurship education and add entrepreneurship nursing to their bachelor’s degree curriculum. This way, the students can learn about entrepreneurship and its role in society. Entrepreneur nurses claim that their training was based on traditional methods and did not provide them with sufficient knowledge about entrepreneurship [21, 22]. Increasing education while serving teachers is another important process. Nursing is a progressive science and art that if stopped, it will appear to have gone backward. In order to improve and integrate nursing profession with advances made in technology, in-service training should be used as a useful and cost-effective method. The headings of continuous education program should also be revised regularly according to the needs of learners.

Quality assurance was another driver of nursing education found in this study. Accreditation in Iran model is more focused on managerial, structural and administrative standards. In a comparative study, Rajai et al. (2022) recommended Canadian model for quality assurance in nursing education of Iran. The Canadian model focuses on collaborative management, inspiring leadership, and development of nursing professional competencies in accordance with the health needs of society. It also emphasizes on the teamwork in various fields of prevention, treatment and rehabilitation, as well as inter-professional education and freedom of action in funding faculty members. With a systemic and holistic approach, this model has presented the standards based on three principles of equality in nursing education, the professional competence of learners, and the capacity of educational institutions, which can be used to improve accreditation in Iran [23].

Each academic environment responds to the globalization needs in a unique way that is a reflection of dominant academic culture in that environment. Nursing education cannot fully protect itself from the effects of globalization in the long run. As a result, isolation and non-participation in the process of globalization cannot be a long-term strategic choice, and sooner or later the country’s nursing education system must prepare itself to face this phenomenon. If the country’s universities can provide their educational and research programs to the applicants according to international standards, the students will be able to easily work beyond the geographical borders by mastering scientific and international languages [24]. Expansion and consolidation of human rights and values such as freedom, justice and equality, reduction in child mortality rate, and also the increase in quality of life and life expectancy are among the positive outcomes of globalization in nursing [25].

Globalization, growth of international exchanges, increases transfer of human resources and immigration have increased the demand for nurses with cultural knowledge. Caring for patients with diverse cultures has been described by nurses as a complex and challenging task. By acquiring cultural competence, the student can obtain the necessary information about the cultural, social and moral status of patient [26]. In addition to culture, the process of educating students’ sensitive to professional ethics should be considered as an important principle. Having an educational module on theoretical ethics cannot guarantee the moral competence of nurses, so ethics in addition to theoretical/practical module, must be taught as a hidden curriculum during the entire program. Also, all educational methods should be used to help students develop moral competence, critical thinking skills, and professional obligation/performance when faced with ethical issues in daily care. Officials of nursing education system should increase the moral development of students by formalizing a moral education program in the form of theory and clinical practice. Also, the problems that exist in the field of ethics education in our country (shortages of specialized tutors in the field of ethics and the lack of a coherent program) should be minimized with coherent planning [27].

### Limitations

One of the limitations of this study is that it only used the opinions of nursing professors. To delve deeper into the experiences of nursing professors, an attempt was made to analyze the experiences in several stages and with several methods. It would be better to use the opinions of nursing students and nurses in future studies.

## Conclusion

If training is designed and implemented without considering drivers and trends, it will lead to a waste of valuable resources. On the other hand, if training is done based on the motivations and educational trends, it will ultimately increase the effectiveness and efficiency of human resources, reduce the waste of valuable resources, develop knowledge and skills (cognitive, technical and human), and increase satisfaction and motivation. In order to control the challenges of nursing education and prevent them from turning into crisis, operational and basic solutions are suggested to be used to continuously improve and upgrade curriculum in accordance with global standards and advances in knowledge and technology. We also need to modify and reduce the tutor/students ratio, establish an accreditation body at national level, and allocate performance-based budget in order to develop education system qualitatively.

## Electronic supplementary material

Below is the link to the electronic supplementary material.

Supplementary Material 1

## Data Availability

The data that support the findings of this study are available from the authors but restrictions apply to the availability of these data, which were used under license from the National Center for Strategic Research in Medical Education (Tehran) for the current study, and so are not publicly available. Data are, however, available from the authors upon reasonable request and with permission from the National Center for Strategic Research in Medical Education (Tehran, Iran).
