# Supplementary material for: Identifying the drivers and trends of Iran nursing education: a multi-methods study
Source: BMC Nurs. 2025 Jul 1;24:712. doi: 10.1186/s12912-025-03169-8 (PMC12210801; doi:10.1186/s12912-025-03169-8)
Supplement: Supplementary file 1 — Supplementary Material 1 [file 12912_2025_3169_MOESM1_ESM.docx]

**Appendix 1**

**Interview guide:**

What are the driving forces of nursing undergraduate education in your opinion?

What are the driving trends of nursing undergraduate education in your opinion?

What are the effective factors on these driving trends?

What factors strengthen these driving trends?

What factors weaken these driving trends?

Please explain more?

Please continue…

Please provide an example.

**Appendix 2**

**The Shannon entropy (weight of concept)^[[1]](#footnote-1)^:**

Equation 1:


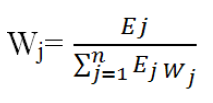


Equation 2:


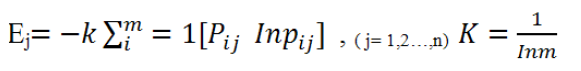


1. Kreiner WA. First Digits’ Shannon Entropy. Entropy. 2022 Oct 3;24(10):1413. DOI:[10.3390/e24101413](http://dx.doi.org/10.3390/e24101413) [↑](#footnote-ref-1)
